# Acquiring Metastatic Competence by Oral Squamous Cell Carcinoma Cells Is Associated with Differential Expression of **α**-Tubulin Isoforms

**DOI:** 10.1155/2012/491685

**Published:** 2012-06-07

**Authors:** Becky Lou, David Engler, William Dubinsky, Jean Wu, Nadarajah Vigneswaran

**Affiliations:** ^1^New York Medical College, 40 Sunshine Cottage Road, Valhalla, NY 10595, USA; ^2^The Methodist Hospital Research Institute, 6670, Bertner Street, Houston, TX 77030, USA; ^3^Department of Diagnostic and Biomedical Sciences, The University of Texas School of Dentistry at Houston, 7500, Cambridge Street, Houston, TX 77054, USA

## Abstract

We performed comparative global proteomics analyses of patient-matched primary (686Tu) and metastatic (686Ln) OSCC cells. The metastatic OSCC 686Ln cells showed greater *in vitro* migratory/invasive potential and distinct cell shape from their parental primary 686Tu cells. Ettan DIGE analysis revealed 1316 proteins spots in both cell lines with >85% to be quantitatively similar (<2 folds) between the two cell lines. However, two protein spots among four serial spots were highly dominant in 686Ln cells. Mass spectrometry sequencing demonstrated all four spots to be **α**-tubulin isotypes. Further analysis showed no significant quantitative difference in the **α**-tubulin between the two cell lines either at mRNA or protein levels. Thus, two distinct isoforms of **α**-tubulin, probably due to posttranslational modification, were associated with metastatic 686Ln cells. Immunofluorescence demonstrated remarkable differences in the cytosolic **α**-tubulin distribution patterns between the two cells. In 686Tu cells, **α**-tubulin proteins formed a normal network composed of filaments. In contrast, **α**-tubulin in 686Ln cells exhibited only partial cytoskeletal distribution with the majority of the protein diffusely distributed within the cytosol. Since **α**-tubulin is critical for cell shape and mobility, our finding suggests a role of **α**-tubulin isoforms in acquisition of metastatic phenotype and represents potential target for therapeutic intervention.

## 1. Introduction

Oropharyngeal squamous cell carcinoma (OSCC) ranks among the top ten most frequent cancers worldwide. Despite recent advances in cancer treatments, the 5-year survival rate for OSCC patients has not changed significantly over the past 30 years, remaining at 30–50%. Most patients with OSCC have metastatic disease at the time of diagnosis, and unlike other malignancies, OSCC spreads exclusively via lymphatic routes to the cervical nodes [1]. The 5-year survival rate is <50% even in patients with a single unilateral lymph node metastasis [1]. Therefore, cervical lymph node metastasis is the major determinant of survival in patients with OSCC. Metastasis which is defined as the spread of tumor cells from the primary site to other tissues of the body is a complex process, involving invasion into adjacent tissues, intravasation, arrest within the microcirculation, and establishment of new growth in regional lymph nodes and/or distant organs [2–4]. To acquire these properties, tumor cells undergo selective changes in their gene expression patterns, leading to overexpression of metastasis-promoting molecules [5, 6]. Recent gene expression profiling studies have demonstrated that cancer's natural history, including metastasis and response to treatment, is indeed encoded in the primary tumors [6–8]. Furthermore, these studies have documented that specific gene expression patterns of primary tumors are better than the routine clinic-pathologic indicators in predicting metastasis, recurrence, and the patient's overall survival.

Tumor cells acquiring metastatic phenotype exhibit specific molecular alterations that regulate cell motility and adhesion, the epithelial to mesenchymal transition (EMT), and extracellular matrix remodeling. Because development regional lymph node metastasis causes much of the morbidity and incurability of OSCC, there is a critical need to identify molecules that confer metastatic phenotype in OSCC [1]. Although there are various methods that have been used to identify molecules involved in metastasis, one of the most convincing approaches is a comparative proteomic analyzes of patient-matched primary and metastatic tumor cells. Identification-metastasis-associated proteomic aberrations are critical for the development of new diagnostic tests for prediction and detection of OSCC metastasis and formulate treatment strategies for OSCC metastasis.

Previously, we reported the gene expression signature that distinguishes primary and metastatic tumor cells derived from the same patient [9, 10]. Here, we extend our study to characterize the proteomic signature of metastatic OSCC cells. We identified a qualitative and qualitative difference in the expression pattern of *α*-tubulin isoforms in metastasizing cells which correlates with their increased motility *in vitro*.

## 2. Materials and Methods

### 2.1. Cell Lines

Details related to primary OSCC (MDA686Tu) and metastatic OSCC (MDA686Ln) cell lines have been described previously [9]. Briefly, the MDA686Tu (686Tu) and MDA686Ln (686Ln) cell lines were established simultaneously from the primary tumor and lymph node metastasis of OSCC (T3N3B) involving the left base of the tongue of a 49-year-old male. These cell lines were maintained in DMEM/F12 50/50 mix (Cambrex) containing 10% fetal bovine serum (FBS), 0.4 *μ*g/mL hydrocortisone, and penicillin-streptomycin-amphotericin antibiotic mix. NIH 3T3 cells (ATCC, Manassas, VA) were grown in DMEM with 10% fetal calf serum (FCS) up to 90% confluent. The medium was removed and replaced by FBS-free DMEM medium supplemented with 50 mg/mL ascorbic acid and cultured for 24 hrs. The supernatant was collected and designated as conditioned medium and used in cell motility and invasion assays.

### 2.2. Tumor Cells Motility and Invasion Assay

A modified Boyden's chamber method using BD Falcon Cell Culture Inserts (BD Biosciences Discovery Labware Franklin Lakes, NJ) is used to determine the *in vitro* tumor cells motility and invasion. The BD Falcon Cell Culture Inserts containing PET (polyethylene terephthalate) membranes (8 *μ*m pore size) without and with Matrigel coating were used for motility and invasion assays, respectively. The 686Tu and 686Ln cells were harvested and resuspended into serum-free medium. The upper chambers of the inserts were filed with 500 *μ*L of serum-free media containing 4 × 10^4^ of cells. NIH 3T3-conditioned medium, mixed with the same volume of DMEM-F12 with 10% FCS, was added to the lower chamber as chemoattractant. The NIH-3T3 conditioned medium was derived from NIH 3T3 cells grown for 24-hours in serum-free DMEM media with 50 mg/mL ascorbic acid. The plates were incubated in a humidified environment at 37°C with 5% CO_2_ for 24 h. At the end the incubation, cells were scraped from upper surface of the membrane by wiping with a cotton swab. The cells adherent to lower surface of the membrane (cells that migrated/invaded) were fixed in 2% of paraformaldehyde for 10 minutes at 4°C and stained with hematoxylin and eosin (H&E). Digital images of the stained cells were obtained from each inserts and used for cell counting.

### 2.3. Ettan DIGE Electrophoresis and Peptide Sequencing

The proteins were extracted from cell lines and quantitated with BCA protein assay kit (Pierce). One hundred *μ*g of proteins from 686Tu and 686Ln were labeled by 1.0 nmol Cy3 and Cy5 respectively following manufacture instruction (Amersham Biosciences). The two labeled samples were combined and further mixed with 2x loading buffer for isoelectric focusing (IEF, 7 m urea, 2 M thiourea, 2% CHAPS, 50 mM DTT and 1% Phamalyte (pH3–10NL). The first-dimension IEF on a rod gel (12 cm × F1.0 mm, pH3–10) was performed under a programmed gradient 200 V to 1,500 V over 18 hrs (total 25 kVh). The first-dimensional gel was equilibrated in 1x SDS-PAGE loading buffer for 3 hrs and loaded onto an isocratic slab SDS-PAGE gel (8–15%) for electrophoresis under constant current of 20 mA overnight. The SDS-PAGE gels were imaged by sequential fluorescence emission spectroscopy using a Typhoon 9400 laser scanner (GE Healthcare, Piscataway, NJ) for each of the spectrally resolvable Cy3/5 dyes (Cy3, 532 nm/580 [bp ± 15] nm; Cy5, 633 nm/670 [bp ± 15]) and digital images recorded using ImageQuant (GE Healthcare) software. Individual protein spots observed within each of the 686Ln (Cy3) or 686Tu (Cy5) images were then quantified and compared after volumetric rendering using the differential in-gel analysis (DIA) algorithm within DeCyder (v5.5; GE Healthcare) software. Individual spots of interest that exhibited a statistically significant difference in volume between the two cell lines were identified. In a conventional 2D electrophoresis under the same conditions, the interested spots were picked after Coomassie blue staining for further mass spectrometry. Mass spectral analyses were performed in the Translational Proteomics Core at UTHealth. Briefly, the proteins were *in-gel* digested as previously described (http://msf.ucsf.edu/ingel.html) [11]. The tryptic digests were taken to dryness in a Thermo SpeedVac and dissolved in 20 uL of 2% acetonitrile, 0.1% formic acid (solvent A). Aliquots of the digest were analyzed by LC/MS/MS on an Agilent 6538 UHD Accurate-Mass Quadrupole Time-of-Flight (Q-TOF) mass spectrometer equipped with an Agilent 1260 nano-LC system. The reverse phase chromatography was performed on Agilent High Capacity Chip (143 mm) using solvent A as initial mobile phase and varying percentages of solvent B (90% acetonitrile, 0.1% formic acid) to constitute a five-stage gradient elution (5–30% B for 28 mins; 30–40% B for 2 mins; 40–90% B for 2 mins; 90% B for 2 mins; 90–3% B for 2 min). Electrospray ionization was operated at the spray voltage of 1.75 kV. Mass spectral data was extracted with MassHunter Quantitative Analysis package and peptides were identified from MSMS spectra with MASCOT. The MASCOT search was performed with a peptide tolerance of 5 ppm and an MSMS tolerance of 0.05 Da, fixed modification was carbamidomethyl and variable methionine oxidation. Identification of nontryptic fragments was performed manually with an initial search on the basis of predicted peptide masses of all possible fragments resulting from novel cleavage sites in the hinge region. The MSMS spectra of suspect peaks were verified by manual *de novo* peptide sequencing to confirm their identities.

### 2.4. Immunofluorescence

Cell suspensions were seeded to each well of 8-well Chamber slides (Fisher Scientific, Rochester, NY) for overnight at 37°C, 5% CO2. The cells were fixed and permeabilized with a kit (Cytofix/Cytoperm, BD, San Jose, CA). After thorough wash with cold PBS, the cells were first incubated with anti-CD32 antibody and normal goat serum for prevention of nonspecific binding of mouse or goat IgG. The cells were then incubated with mouse monoclonal antibody to *α*-actin (clone AC-40, Sigma, St. Louis, MO) or *β*-actin (clone AC-1, Sigma, St. Louis, MO), followed incubation with FITC-labeled goat anti-mouse IgG (Southern Biotech, Birmingham, AL). The cells were further stained by with Alex(R) 555-labelled rabbit monoclonal antibody against *α*-tubulin antibody (Cell Signaling Technology, Beverly, MA). Finally, the cells were counter stained with DAPI. The cells were observed under a fluorescent microscope (Eclipse 80i, Nikon, Tokyo, Japan). The digital images were recorded and analyzed by NIS-Elements (Nikon Image System).

### 2.5. Statistical Analysis

Two-sample *t*-test and one-way ANOVA were used to determine the statistical significance of the differences in migration and real-time PCR between 686Ln and 686Tu lines. Statistical analyses for DIGE were performed using ImageQuant software from GE Health.

## 3. Results and Discussion

### 3.1. Higher Invasion Potential of 686Ln Cells Isolated from Regional Lymph Nodes

**Figure 1:**
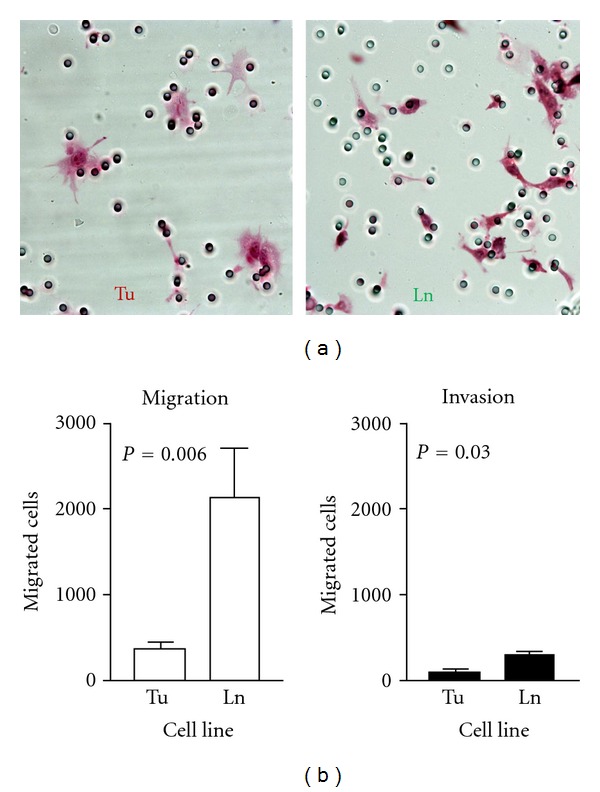
Metastatic OSSC 686Ln cells have higher invasion potentials than primary 686Tu cells. (a) Morphology of migrated OSSC 686Tu (Tu, left panel) and 686Ln cells (Ln, right panel) on the filter after H-E staining during migration assay. Pores on the filter are visible: ×300. (b) Summary of invasion potentials of 686Tu (Tu) and 686Ln (Ln) cells in migration and invasion assays as indicated at the top of each graph. Invasion potentials are expressed as numbers of migrated cells in each well.

Previously, we analyzed the global gene expression profiles of 686Tu and 686Ln and demonstrated that expression levels of >90% of the genes in cell lines derived from the primary and metastatic tumors of the same patient were identical to each other than the two metastatic cell lines from two different patients [9]. In line with the published reports, our study confirmed that the gene expression pattern of primary tumor cells are mostly preserved in their metastatic counterpart except for a few differentially expressed genes which are implicated in promoting metastasis [9, 12]. We performed cell motility and invasion assays to characterize the phenotypic differences between 686Tu and Ln cells. In the cell motility assay performed using the cell culture insets without Matrigel coating, 686Ln cells revealed enhanced migration which was 5.5 fold higher than that of 686Tu cells (left panel in Figure 1(b)). In the invasion assay performed using the cell culture inserts coated with three-dimensional Matrigel, invasion of both cell lines were greatly decreased. However, the reductions rates were significantly different between 686Tu and 686Ln cells. The 686Ln cells showed only 3-fold reduction as compared to 686Tu cells with 7.1-fold reduction (Figure 1(c)). As a result, only a 3-fold difference was observed between 686Ln and 686Tu cells when invading through Matrigel, as compared to 5.5-fold difference when migrating through noncoated membrane. This data suggests that the molecular machinery that underlies cell motility is more critical for metastatic phenotype than their extracellular matrix degrading molecules which are essential for invasion through the Matrigel. Tumor cells are known to change their phenotype during culture. However, our result showed that the metastatic 686Ln cells demonstrate higher motility and invasive potential than their parent primary tumor cells and indicate that the phenotypic features acquired *in vivo* are also preserved in cultured cells. Therefore, this pair of cell lines represents an ideal model to characterize proteomic signature that is causal for metastatic phenotype.

### 3.2. Qualitative and Quantitative Differences in *α*-Tubulin Expression Patterns between 686Tu and 686Ln Cells are Caused by Posttranslational Modifications

**Figure 2:**
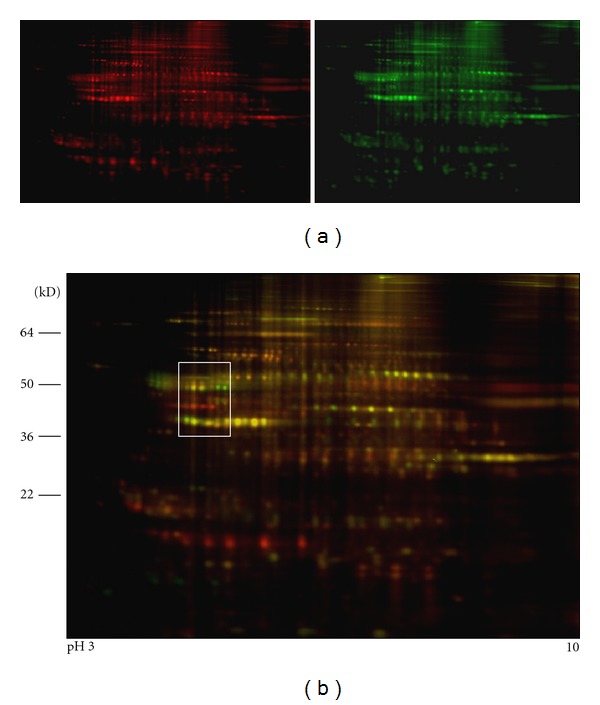
DIGE gel images of primary OSSC 686Tu and metastatic 686Ln cells. (a) Cy5 image (red, left panel) and Cy3 image (green, right panel) for 686Tu (Tu) and 686Ln (Ln), respectively. (b) Merged Cy5 and Cy3 image. Rectangular area, outlined by white line, would be further discussed. Note that some spots in the area appear pure green or red color, suggesting their uneven quantities in the two cells.

**Figure 3:**
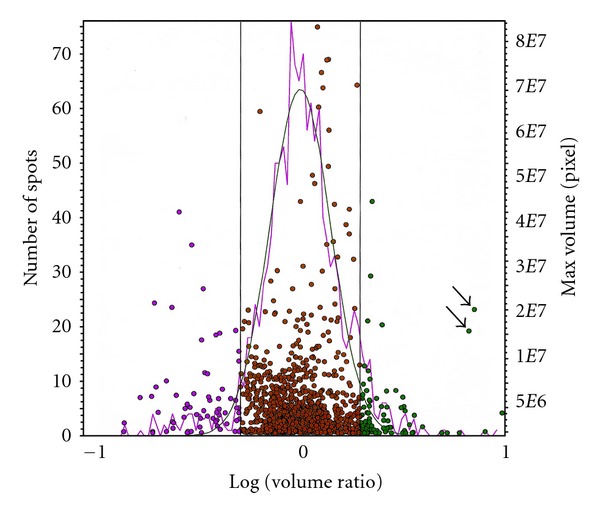
Scattergram depiction of DIGE data to generate protein expression profile of 686Tu and 686Ln cells. Each dot represents a protein detected in 686Tu and 686Ln cells. The dots are expressed as bigger pixel volume in either cell (right *Y*-axis), and plotted against log scale of pixel volume ratio between 686Tu and 686Ln (*X* axis), with 686Tu as standard. Two vertical lines indicate two-fold difference limits between the two cells. Left *Y*-axis is number of spots at certain pixel ranges. Related pink curve is actual distribution of spots versus their pixel volume ratio and green curve is normalized Gaussian distribution (*R* ^2^ = 0.963). Arrows indicate two protein spots to be studied.

 Proteins from the two cell lines were used for DIGE 2D gel electrophoresis. The scanned Cy3 and Cy5 images for 686Ln and 686Tu, respectively, displayed similar spot distribution patterns (Figure 2(a)). In line with our gene expression profiling data, the metastatic OSCC cells and its parent primary tumor cells revealed almost identical cellular protein profiles in CyDy DIGE analysis (Figure 2(a)). Based on our previous testing, over 5-fold difference in a protein of two sources would generate obvious green or red spot. In combined image, dots with obvious green or red color were visible (Figure 2(b)). Squared area in Figure 1(b) is an example to show different colors. With 2 × 10^4^ pixels as background, the software detected 1316 protein spots in combined image for 686Tu (Cy5) and 686Ln proteins (Cy3), see Figure 3. Pixel volume for each spot was calculated and compared between 686Ln and 686Tu cells using 686Tu as standard. Plot with log volume ratios against spot numbers fit well with Gaussian distribution (*R* ^2^ = 0.9629) with 0.032 ± 0.16 in Log scale or 1.033 ± 1.176 folds (Figure 3). To estimate how closely the two cell lines were related, we used 2-fold differences in pixel volume as threshold. Calculation demonstrated that 686Ln and 686Tu cells showed similar volumes in 1159 spots (88.1%), suggesting their close relationship. In addition, 686Ln cells showed increased volume in 70 spots (5.3%) and decreased volume in 87 spots (8.6%), as compared to those of 686Tu cells. However, most spots with different volumes between the two cells clustered between 2 to 4 folds (Figure 3).

In this study, we solely focused on the protein spots, which were significantly increased in 686Ln. Plot of log volume ratio versus max volume revealed two protein spots which showed more than 5-fold increase in 686Ln cells with volumes higher than 2 × 10^7^ pixels (arrows in Figure 3). These two protein spots were identified to be two green spots in an acidic region (pH5–5.5) with molecular weight 40–60 kD (squared area in Figure 1(b)). These two green spots were among series of 5 dots with similar molecular masses of 55 kD. The two green spots migrated to higher pH region (pH5.5). Those 5 proteins were designated as number 1 to 5 with number 1 and 2 for the two green spots (Figure 3(a), arrows in Figure 3). Calculation of pixel volumes demonstrated that number 1 and 2 in 686Ln cells were 7.5 and 7.1 folds of those in 686Tu cells. On the other hand, volume ratios between 686Ln and 686Tu cells were 1.02, 0.76, and 0.69 for spots number 3, 4 and 5, respectively. At least three spots in the adjacent area were found to be greatly increased in 686Tu cells (red, numbered as number 6–8, Figures 2 and 3). However, we did not explore the identities of these spots in this study.

**Figure 4:**
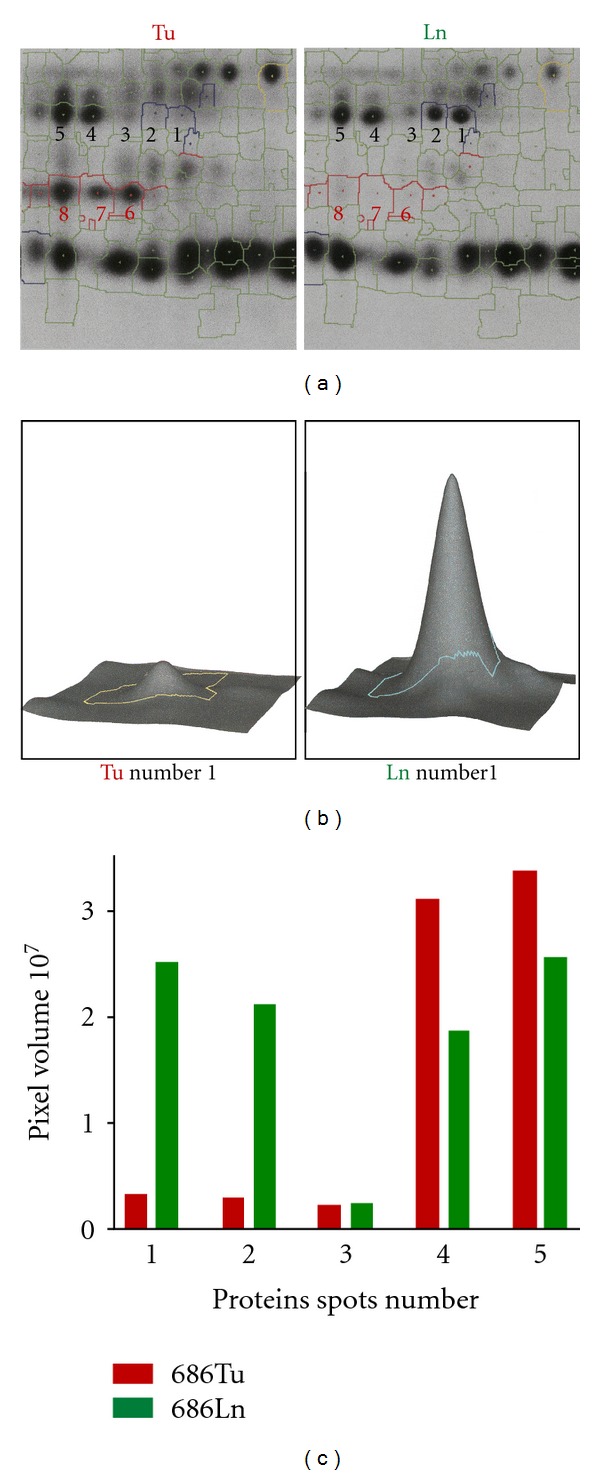
Comparison of protein spots between 686Tu and 686Ln cells in rectangular area (see Figure 1(b)). (a) Enlarged images of interested area for 686Tu (Tu, left panel) and 686Ln cells (Ln, right panel). Some spots are numbered. Note that spots number 1 and 2 are absent in 686Tu cells, while number 6, 7, and 8 are missing in 686Ln. (b) 3D simulation of spot number 1 in 686Tu and 686Ln cells. (c) Comparison of pixel volumes of spots number 1 to 5 between the two cells. Pixel volumes are calculated with 3D simulation.

**Figure 5:**
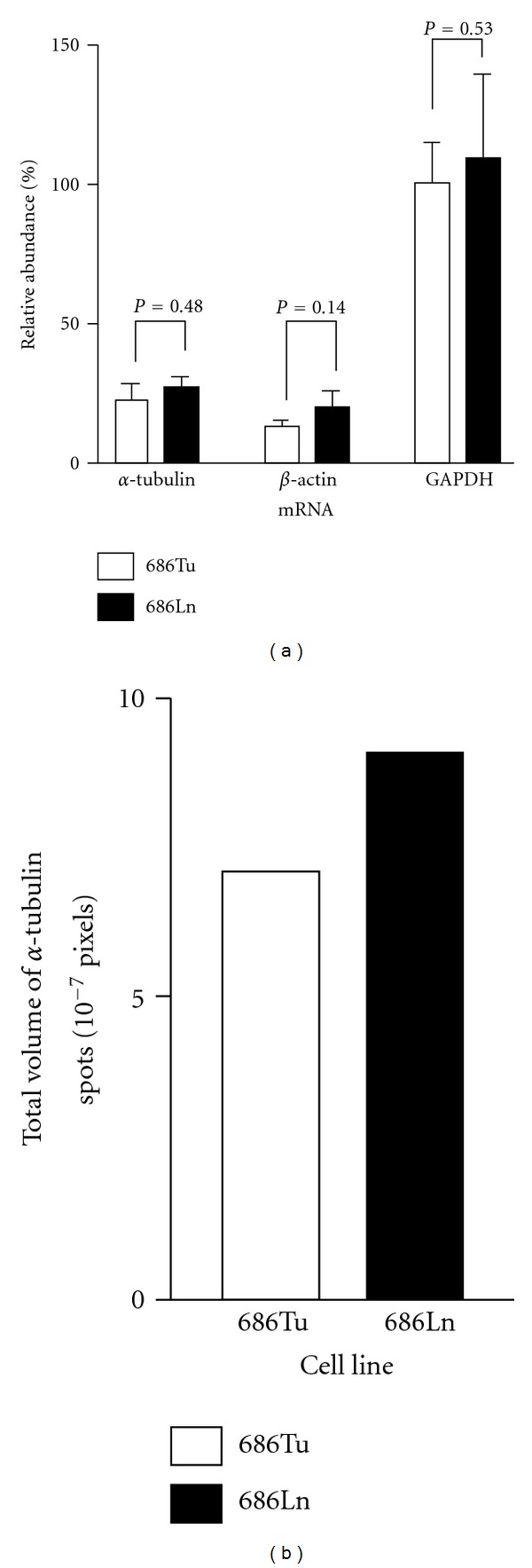
686Tu and 686Ln cells expressed comparable levels of *α*-tubulin. (a) Quantitative RT-PCR detection of mRNA for the genes as indicated in 686Tu and 686Ln cells. The quantities of mRNA are expressed as relative abundance using GAPDH in 686Tu as 100%. (b) Total pixel volumes of *α*-tubulin in 686Tu and 686Ln cells.

We next performed a routine preparative 2D electrophoresis of proteins from 686Ln cells (Figure 4). Based on the spots distribution pattern, spots number 1, 2, 4, and 5 were excised for mass spectrometry based peptide sequencing (Figure 4). Spot number 3 was not tested due to its low quantity. Sequencing analyses demonstrated that spots number 1 and 2 were *α*-tubulin. Multiple proteins, including *α*-tubulin, *β*-tubulin, and others, were detected in spots number 4 and 5, probably due to their merge with protein spots of slightly higher mass (see Figure 3(a)). However, the majority of peptides matched to *α*-tubulin. Our previous microarray data showed that expression levels of *α*-tubulin transcripts are not significantly different between 686Tu and Ln cells whereas *β*-tubulin is one of the highly expressed genes in metastatic OSCC cells [9]. To further confirm the microarray data on *α*-tubulin, we performed quantitative real-time RT-PCR analysis. Quantitative RT-PCR demonstrated that copy numbers of *α*-tubulin mRNA in 686Ln cells are marginally higher than 686Tu cells but the difference was not significant (Figure 5). When we calculated the sums of four spots representing the *α*-tubulin protein in these cell lines, we found that total *α*-tubulin protein amount was increased by 1.27 folds in 686Ln cells (9.01 × 10^7^ pixels, 1.27-fold) compared to 686Tu cells (7.08 × 10^7^ pixels, see Figure 5). Although the above data revealed only marginal increase in *α*-tubulin expression at both mRNA and protein levels in metastatic cells compared to its parent primary tumor cells, this small quantitative difference is unlikely to play a causal role in their motility differences.

**Figure 6:**
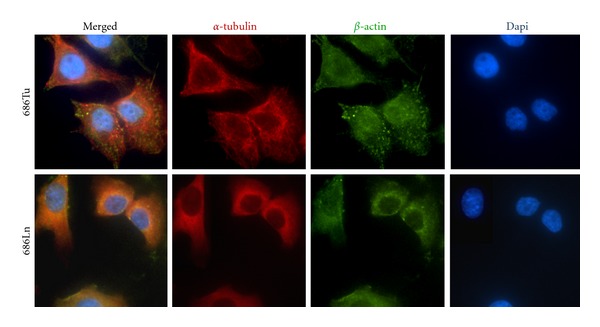
Metastatic 686Ln and primary 686Tu cells show a different cellular distribution pattern of *α*-tubulin. 686Tu (Tu, upper row) or 686Ln cells (Ln, lower row) were stained for *α*-tubulin (red) and *β* actin (green). The cells were counterstained by DAPI. Note that 686Ln cells show defuse *α*-tubulin distribution, in contrast to filament network in 686Tu cells ×600.

Next, we examined whether the immunofluorescence staining patterns of *α*-tubulin protein in this pair of cell lines would reveal any remarkable quantitative and/or qualitative differences. Overall, 686Ln cells showed a higher intensity of anti-*α*-tubulin immunofluorescence staining compared to 686Tu cells (Figure 6). Both cell lines demonstrated a higher density of *α*-tubulin expression in their perinuclear region. A comparison of the staining patterns of *α*-tubulin in 686Tu and Ln cells showed significant qualitative differences in the *α*-tubulin filament distribution. In 686Tu cells, *α*-tubulin proteins formed a network composed of coarse filaments, probably associated with cytoskeleton. Pattern of this network was similar to those in many other cell types including nontransformed cells [13]. In contrast, *α*-tubulin in 686Ln cells exhibited only partial cytoskeletal distribution with majority the protein diffusely distributed within the cytosol. As a result, a uniform filament network as seen in 686Tu cells was not evident in 686Ln cells (Figure 6). Examination of the cells under higher magnification revealed that the cytoskeleton of 686Ln cells is composed of a network of much finer *α*-tubulin filaments (Figure 6). It should be noted that the 686Tu and Ln cells exhibit distinguishable morphologies, especially after migrating through the barrier membrane during motility and invasion assays. Almost all 686Tu cells displayed a round-flattened shape (Figure 1(a)). In contrast, more than 80% of 686Ln cells were elongated and branched, with only a small fraction to be in round spread shape (Figure 1(a)). Hence, we asked whether the unusual *α*-tubulin network in 686Ln cells was due to overlapping of multiple layer of the network because of their elongated and rounded morphology. We selectively examined a number of 686Ln cells with flattened morphology and found that none of these cells exhibited *α*-tubulin staining pattern similar to that of 686Tu cells. This finding suggests that the observed difference in the *α*-tubulin networks between 686Ln and 686Tu cells was not an artefact related to the cellular morphology. Moreover, we compared the immunofluorescences staining patterns of *α*-actin and *β*-actin in these two cell lines and showed that their staining patterns are indistinguishable between 686Tu and 686Ln cells (Figure 6). The sizes of *α*- and *β*-actin filaments are similar between these two cell lines, except for minor differences in their distribution patterns. These findings strongly suggest that the altered *α*-tubulin filament distribution pattern in 686Ln cells is most likely due to its posttranslational modification resulting in altered polymerization and/or increased solubility.

Our proteome analysis confirms that two distinct isoforms of *α*-tubulin are found in higher quantity in 686Ln than in 686Tu whereas the other two *α*-tubulin isoforms are not. Moreover, there is no significant quantitative difference either in the *α*-tubulin transcripts number or total protein levels between these two cell lines. It raises an interesting question whether these two distinct *α*-tubulin isoforms detected in 686Ln cells are unique for metastatic phenotype. Although our study does not provide direct evidence to support that the differences in the *α*-tubulin isoforms and distribution pattern in 686Ln cells are causative for metastasis, published reports have suggested a critical role for tubulin isoforms in tumor cells motility and metastasis.

 Microtubules, a major component of cytoskeleton are involved in various cellular processes such as cell division, intracellular organelle trafficking, secretion, maintenance of cell shape, and motility. Tubulin (100 kDa of two subunits), a major component of microtubules, is a heterodimer of *α*- and *β*-tubulin isotypes which are translated from seven genes [14]. Differential expression of *α*-and *β*-tubulins isotypes are associated with tumorigenesis, metastasis, and resistant to chemotherapy [15–18]. Increased expression of class IV *β*-tubulin isotype in breast cancer cells confer them increased resistance to docetaxel [16]. Docetaxel and paclitaxel are antimitotic chemotherapy drugs used for treating metastatic breast cancer. These drugs bind to *β*-tubulin and halt cell division at metaphase by preventing spindle assembly [19]. In contrast to *β*-tubulin, *α*-tubulin isotypes undergo significant posttranslational modifications that determine microtubule assembly, distribution pattern, and cellular phenotype. Polyglutamylation and detyrosination of *α*-tubulin are frequently noted in prostate cancer cells but not in nontransformed prostate epithelial cells [20]. Increased expression of acetylated of *α*-tubulin is specific for aggressive hormone-refractory prostate cancer cells [17].

Although it is not clear whether tubulin is directly involved in metastatic process, recent studies suggest that abnormal assembly of tubulin monomers may be associated with tumorigenic and metastatic phenotypes [15–17, 21–25]. Several potential mechanisms have been proposed. Microtentacles formed by tubulins in breast cancer cells accelerated cell detachment from ECM, which was a necessary step for metastasis [26–28]. Active disassociation and re-assembling of tubulins promotes cancer cells motility [29].

It has been shown that the cell motility is modulated by *α*-tubulin acetylation/deacetylation by impacting the assembly and disassembly of microtubules [30, 31]. Although the enzyme responsible for *α*-tubulin acetylation is yet to be identified, deacetylation of *α*-tubulin is catalyzed by histone deacetylase 6 (HDAC6) [32]. On the other hand, HDAC6 inhibitor blocks tumor cells migration and reduces their metastatic potential by inducing acetylation of *α*-tubulin [33].

 Our findings indicate that the morphologic changes exhibited by 686Ln cells during their *in vitro* migration/invasion are reminiscent of morphologic features associated with epithelial-mesenchymal transition (EMT) in which epithelial cells acquire fibroblast-like properties [34]. Epithelial malignant tumor cells are critically dependent on EMT for their invasion and metastatic spread [34]. Recently, it was reported that transforming growth factor-*β*1- (TGF-*β*1-) induced EMT in tumor cells is indeed mediated by deacetylation of *α*-tubulins by HDAC6 [35]. Previous studies on tubulin family have shown that members of *α*-tubulins have very similar molecule weight and isoelectric point, which make them difficult to be separated even in 2D electrophoresis. Moreover, both tubulin spots specific for 686Ln cells were characterized as *α* tubulin 1C. Based on the published reports and our findings, we propose that two isoforms of *α* tubulin in metastatic 686Ln are caused by posttranslational modification, most likely deacetylation. Diffuse distribution of *α* tubulin in 686Ln suggests relatively unstable microtubules or presence of soluble *α* tubulins due to posttranslational modifications [18, 36]. However, the biochemical features of these posttranslational modifications remain to be explored. Although the findings of this study are encouraging, the data are preliminary based on *in vitro* studies. Hence, this data needs to be confirmed *in vivo* by examining a larger number of patient matched primary and metastatic OSCC tumor sections.

## 4. Conclusion

In conclusion, metastatic OSCC cells exhibit increased *in vitro* motility and Matrigel invasiveness than their parental primary tumor cells derived from the same patient. We identified two distinct *α*-tubulin isoforms specific for metastatic cells that are not present in its parental primary tumor cells. Moreover, *α*-tubulin filaments exhibit distinctly different distribution pattern in metastatic cells compared to its parental primary tumor cells. We, therefore, propose that differential posttranslational modifications of *α*-tubulin are involved in acquiring metastatic phenotype in OSCC and characterization of these specific modifications may open new avenues for therapeutic intervention against OSCC metastasis. However, it remains to be determined whether the differential expression of metastatic phenotype-specific *α*-tubulin isoforms can be demonstrated in patient-matched primary and metastatic tumor specimens.
